# Endogenous tick viruses and modulation of tick-borne pathogen growth

**DOI:** 10.3389/fcimb.2013.00025

**Published:** 2013-07-12

**Authors:** Lesley Bell-Sakyi, Houssam Attoui

**Affiliations:** The Pirbright InstituteSurrey, UK

**Keywords:** tick, endogenous virus, tick cell line, mosquito, pathogen, co-infection, dsRNA virus, St Croix River virus

## Abstract

Ticks transmit a wide range of viral, bacterial and protozoan pathogens, many of which can establish persistent infections of lifelong duration in the vector tick and in some cases are transmitted transovarially to the next generation. In addition many ixodid and argasid tick cell lines and, by inference the parent ticks from which they were derived, harbor endogenous viruses (ETV) of which almost nothing is known. In general, low level persistent infections with viral pathogens (arboviruses) are not known to have a deleterious effect on tick survival and fitness, suggesting that they can strike a balance with the tick innate immune response. This tolerance of arbovirus infection may be modulated by the permanent presence of ETV in the host cell. In mosquito cells, temporary or permanent silencing of the genes of an endogenous virus by RNA interference can result in changes in replication rate of a co-infecting arbovirus. We propose that tick cell lines offer a useful model system for *in vitro* investigation of the modulatory effect of ETV on superinfecting pathogen survival and replication in ticks, using the molecular manipulation techniques applied to insect cells.

## Background

Ticks are haematophagous parasitic arthropods that feed on a wide range of mammalian, avian, reptilian and amphibian hosts. There are nearly 900 known species of ticks of which 193 belong to the Argasidae (soft ticks) and 702 to the Ixodidae (hard ticks) (Guglielmone et al., 2010). As well as causing direct damage to the vertebrate host during feeding (including skin damage, blood loss, and in some cases immunosuppression), around 10% of tick species are known to be vectors of microorganisms causing disease in livestock, companion animals and humans (Jongejan and Uilenberg, 2004). Most of the arboviruses transmitted by ticks are RNA viruses in the families *Flaviviridae, Bunyaviridae*, and *Reoviridae*, including the causative agents of tick-borne encephalitis, Crimean-Congo hemorrhagic fever and Kemerovo tick-borne viral fever infections in humans, and louping ill and Nairobi sheep disease in domestic ruminants (Nuttall, 2009; Belhouchet et al., 2010). The only confirmed DNA arbovirus, African swine fever virus (*Asfarviridae*), is transmitted by ticks of the genus *Ornithodoros* (Plowright et al., 1969), and recent evidence suggests that ixodid ticks may play a role in transmission of the DNA poxvirus causing lumpy skin disease (Tuppurainen et al., 2011). Pathogenic bacteria transmitted by ticks include *Borrelia* spirochaetes and members of the obligately intracellular genera *Anaplasma, Ehrlichia* and *Rickettsia* (Jongejan and Uilenberg, 2004); ticks may also play a role in reservoir maintenance and/or transmission of *Bartonella, Coxiella* and *Francisella* species (Parola and Raoult, 2001; Reis et al., 2011). The life cycle and transmission of the protozoan pathogens *Babesia* and *Theileria* is intimately bound up with the life cycles and development of their tick vectors (Young and Leitch, 1980; Florin-Christensen and Schnittger, 2009).

That ticks harbor apparently endosymbiotic bacteria has been known for many years (Cowdry, 1925), but identification and characterization of many of these bacteria has only become possible with the advent of molecular phylogenetic techniques and development of suitable *in vitro* culture systems. These endosymbiotic bacteria persistently infect all life cycle stages of the ticks and are passed on to the next generation transovarially. It is unclear whether or not bacterial endosymbionts are transmitted to vertebrates during tick feeding; a recent study of humans bitten by *Ixodes* ticks suggests that salivary transmission of the intramitochondrial tick symbiont *Candidatus* Midichloria mitochondrii (Sassera et al., 2006) can occur to a level sufficient to induce production of specific antibodies (Mariconti et al., 2012). In contrast, *Rickettsia peacockii*, an endosymbiont of the tick *Dermacentor andersoni*, was not pathogenic for laboratory animals and failed to infect a range of mammalian cells *in vitro* (Kurtti et al., 2005). Many of these endosymbionts, including *Candidatus* M. mitochondrii and the *Francisella*-like endosymbionts of *Dermacentor* spp ticks, have only been detected by PCR amplification of gene fragments and/or microscopy (Noda et al., 1997; Scoles, 2004; Epis et al., 2013) but a small number have been isolated and propagated *in vitro* in tick cell lines (Kurtti et al., 1996; Simser et al., 2001, 2002; Mattila et al., 2007). Progress in this area is only limited by the number of researchers, their access to infected ticks, and the range of cell lines derived from appropriate tick species.

## Tick cell lines and endosymbiotic bacteria

The first continuous tick cell lines were established from developing adult *Rhipicephalus appendiculatus* ticks nearly 40 years ago (Varma et al., 1975). Thereafter, a combination of improvements in culture methods and, more recently, greatly increased interest in tick cells as research tools, led to the present situation in which over 50 continuous tick cell lines, established from two argasid and fourteen ixodid tick species, are currently in existence (Bell-Sakyi et al., 2007, 2012). The majority of these can be obtained through the Tick Cell Biobank (http://tickcells.pirbright.ac.uk).

Tick cell lines have been applied in many areas of tick and tick-borne pathogen research, including biology, functional genomics, proteomics, antibiotic susceptibility, acaricide resistance and vaccine development (Bell-Sakyi et al., 2007, 2012). Partly due to the availability of a sequenced and partially annotated genome, around 80% of >150 studies published since 1995 have utilized either or both of two particular cell lines, IDE8 and ISE6 derived from embryonic *Ixodes scapularis* (Munderloh et al., 1994; Kurtti et al., 1996). These two cell lines support isolation and growth of numerous intracellular bacteria, including tick-borne pathogens such as *Anaplasma marginale, Anaplasma phagocytophilum, Ehrlichia ruminantium*, and *Ehrlichia chaffeensis* (reviewed by Bell-Sakyi et al., 2007).

Other tick cell lines have themselves been found to harbor originally endosymbiotic bacteria, which may eventually come to dominate the balance with their host cells, resulting in deleterious effects. When the *Dermacentor andersoni* cell line DAE100 was cured of chronic infection with *R. peacockii*, growth rate in the cured cells more than doubled compared to infected cells (Kurtti et al., 2005). Four out of five *Carios capensis* cell lines were found to harbor rickettsial endosymbionts which eventually interfered with cell survival (Mattila et al., 2007).

In two recent studies, bacterial DNA, possibly indicating persistent infections with endosymbionts, was detected in tick cell lines. Najm et al. (2012) intermittently detected a small fragment of a *Candidatus* M. mitochondrii gene in two tick cell lines derived from *Rhipicephalus* (*Boophilus*) *decoloratus* and *Ixodes ricinus*, but were unable to amplify a larger section of a different bacterial gene from the same samples. DNA sequences matching *Rickettsia africae* and *Francisella*-like endosymbionts were amplified from cell lines derived from, respectively, *Amblyomma variegatum* and three different *Dermacentor* species, although no actual bacteria were seen by light or electron microscopy (EM) (Alberdi et al., 2012). It is unclear whether, in the two above-mentioned cases, intact replicating bacteria are present in the tick cell lines or fragments of bacterial genome have become integrated into the host genome as described for *Wolbachia* in woodlice (Martin et al., 2010).

The phenomenon of microbial DNA becoming integrated into the host cell genome is well-recognized by virologists (Feschotte and Gilbert, 2012). In the past, the term endogenous virus has been used to refer to DNA copies of viral genomes integrated into the DNA genomes of hosts/vectors; these endogenous viruses are now generally known as endogenous virus elements (EVE). In practice, the term endogenous virus refers to replicating viruses which have been identified in cells by a variety of techniques including EM, virus isolation and/or molecular techniques. In arthropods some of these replicating viruses are strict arthropod-only viruses, while others can infect vertebrate cells and hence are regarded as actual or potential arboviruses.

## Endogenous viruses in tick cell lines

**Figure 1:**
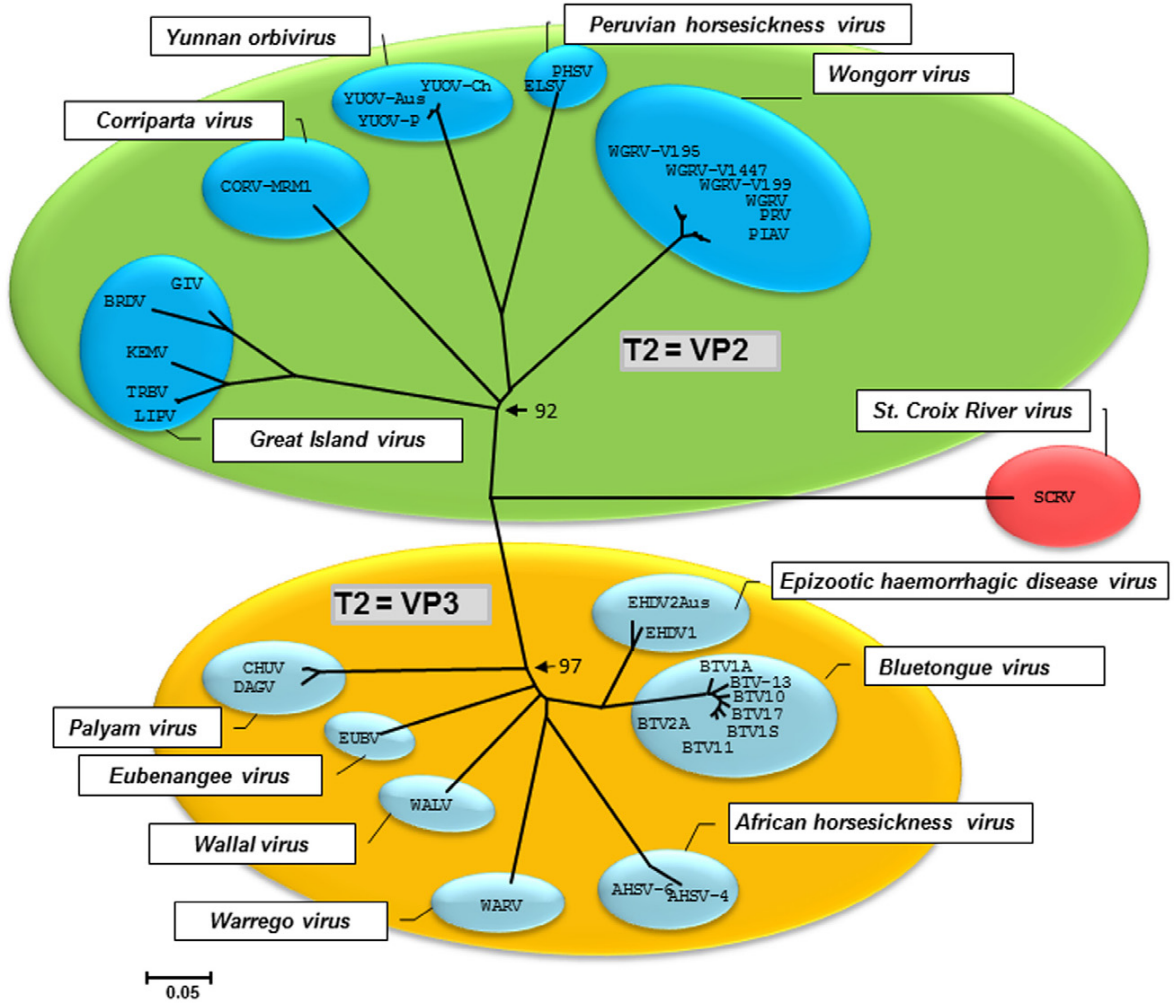
**Neighbor joining phylogenetic tree constructed using the subcore shell protein (T2 protein) of orbiviruses**. The T2 protein of tick-borne and mosquito-borne orbiviruses (green group) is the VP2, while in midge-borne orbiviruses (yellow group) the T2 is VP3. SCRV (red) roots all known orbivirus T2 proteins and represents the ancestral lineage (Attoui et al., 2001). The tree was constructed using the *P*-distance algorithm implemented in the MEGA5 programme. The scale bar indicates the number of substitutions per site. Values at the nodes indicate bootstrap confidence.

It has only recently been recognized that most, if not all, continuous tick cell lines harbor viruses that are presumed to be endogenous. The first of these, named St Croix River virus (SCRV), was detected in the *I. scapularis* cell line IDE2 over a decade ago (Attoui et al., 2001); to date, this orbivirus (*Reoviridae*) remains the only identified and sequenced “tick virus” (Nuttall, 2009; Belhouchet et al., 2010). Phylogenetic analysis indicates that SCRV represents a lineage ancestral to other known tick- and insect-borne orbiviruses (Figure 1). When other *I. scapularis* cell lines were screened by PCR for SCRV, the virus was found in IDE8 but not in IDE12, ISE6 or ISE18 (Alberdi et al., 2012). SCRV was also detected in two *R. appendiculatus* cell lines, RA243 and RA257 (Alberdi et al., 2012) that were established at least 15 years earlier than the *I. scapularis* lines in a different laboratory (Varma et al., 1975) and from ticks originating from a different continent. Although there is now some doubt about the geographic origin of SCRV (North America or Africa?), its “tick only” nature is strongly supported by failure to infect any non-tick cells with this virus including cell lines derived from the mosquitoes *Aedes albopictus, Aedes pseudoscutellaris* and *Aedes aegypti*, the amphibian *Xenopus laevis* (XTC cells) and mammalian Vero, BHK-21, BSR, BGM, KB, HELA, Hep2 cells (authors' unpublished results), indicating that it is highly unlikely to be an arbovirus. On the other hand, SCRV was passaged successfully alongside the bacterial pathogen *Ehrlichia ruminantium* from IDE8 (Bell-Sakyi et al., 2000) to ISE6 cells, passaged 5 weeks and several subcultures later from the ISE6 cells to the *Rhipicephalus sanguineus* cell line RSE8 (Kurtti et al., 1982), and virus was demonstrated in RSE8 cells 2 months later by PCR amplification of a portion of the SCRV segment 2 as described previously (Alberdi et al., 2012), indicating that SCRV can replicate in cells from at least three different tick species (authors' unpublished results).

**Figure 2:**
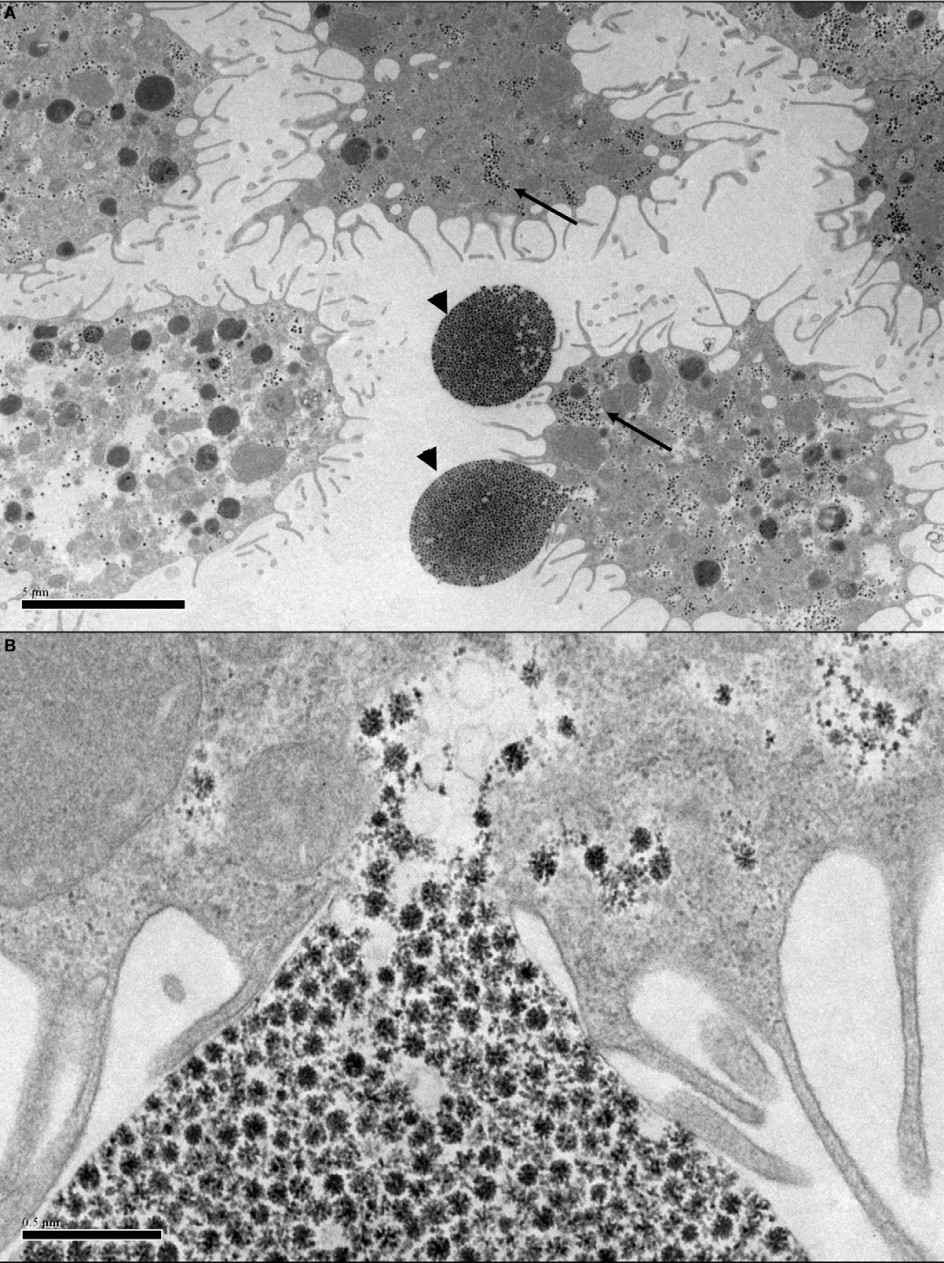
**Transmission electron micrographs of *Ornithodoros moubata* cell line OME/CTVM25 showing putative endogenous viruses. (A)** Cells with “filopodia” extending from the cell surface. Reovirus-like particles (arrows) are abundant in the cytoplasm. The viruses appear to be using the filopodia to form vesicles (arrowheads) carrying a large number of virus particles with diameters of 75–80 nm that may be budding from the cells into the supernatant medium. Scale bar 5.0 μm. **(B)** Closer view of **(A)** showing the site of attachment of the virus vesicle to the cell membrane. Scale bar 0.5 μm.

**Figure 3:**
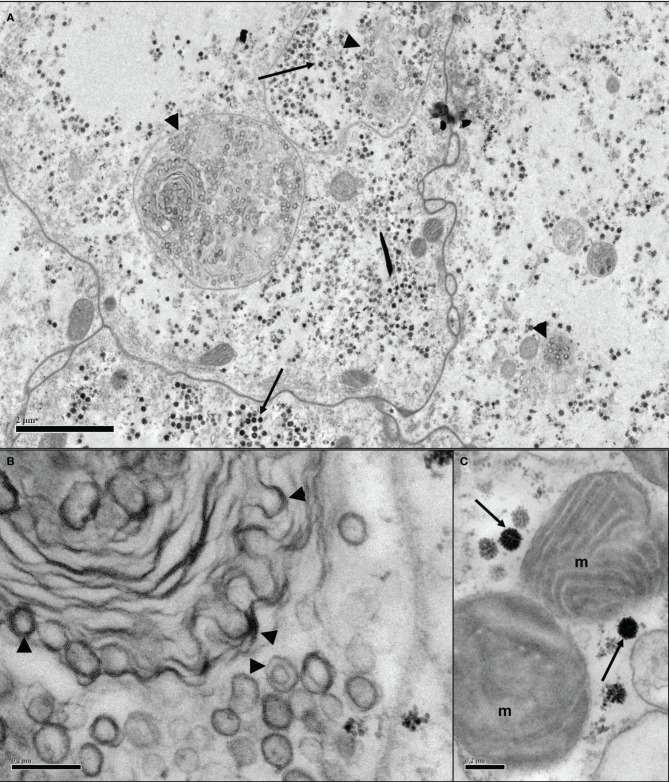
**Transmission electron micrographs of *Ornithodoros moubata* cell line OME/CTVM26 showing putative endogenous viruses. (A)** Several adjoining cells containing putative reovirus-like (arrows) and bunyavirus-like (arrowheads) particles in both individual and shared cellular compartments. Scale bar 2.0 μm. **(B)** Bi- and tri-layered bunyavirus-like particles (arrowheads) appear to be budding from intracellular membranes of unknown function. Scale bar 0.2 μm. **(C)** Icosahedral virus particles of around 100 nm diameter (arrows) in close proximity to mitochondria (m). Scale bar 0.2 μm.

Alberdi et al. (2012) examined 35 ixodid tick cell lines by EM and found that at least 25 of them, including IDE2 and IDE8, contained reovirus-like particles, while out of 47 ixodid and argasid cell lines screened by PCR for SCRV, only the four mentioned above were positive. The eight argasid cell lines included in that report were not examined by EM, but all have since been examined and found to harbor large numbers of icosahedral virus particles (Figure 2), the structure of many of which is reminiscent of reoviruses. In some lines, structures suggestive of bunyaviruses (Figure 3) have also been observed (authors' unpublished data). Putative novel nairovirus (*Bunyaviridae*) sequences were amplified by PCR from several of these argasid lines, as well as from nine of the ixodid cell lines (Alberdi et al., 2012). Thus, it appears that endogenous viruses are commonly present in tick cell lines and, by inference, in the parent ticks from which the cell lines were derived; certainly viruses other than SCRV present in the ~90% of cell lines that are derived from tick embryos must have been transovarially transmitted. However, to date no reports have been published of examination of whole ticks or tick tissues for presence of SCRV or any other putative endogenous virus; such a study involving both *I. scapularis* and *R. appendiculatus* ticks collected from the field might answer the question of the origin of SCRV. On the other hand, if the structures described as “glycogen granules” in early EM studies of tick tissues could actually have been endogenous viruses, as inferred by Alberdi et al. (2012), there exists visual evidence of their distribution in ixodid tick salivary glands (Fawcett et al., 1982), perineurial cells (Binnington and Lane, 1980) and midgut cells (Jaworski et al., 1983). Of particular interest are two EM studies of argasid tick Malpighian tubules in which accumulations of “glycogen particles” are seen both within the cytoplasm of pyramidal and cuboidal cells and packed into apical extensions apparently budding out from the surface of pyramidal cells (El Shoura, 1987, 1988). Their resemblance to the structures seen in *O. moubata* cell lines *in vitro* (Figure 2) is striking. Whether or not the endogenous viruses detected in tick cell lines are “tick only” can only be definitively determined by exhaustive *in vitro* infection studies using cell lines derived from other arthropods, other invertebrates and vertebrates, and *in vivo* inoculation of potential vertebrate hosts. However, partial sequence determination and comparison with other viruses within the same genus may yield clues as to the ability of these novel viruses to infect cells derived from different host taxa.

The genus *Orbivirus* encompasses arboviruses transmitted to vertebrate hosts by mosquitoes, midges and ticks, as well as the apparently “tick only” SCRV. Until recently, the genome of orbiviruses was believed to encode 7 structural proteins (VP1–VP7) and 3 non-structural proteins (NS1–NS3). Belhouchet et al. (2011) identified a novel non-structural protein (NS4) that is encoded by an alternate open reading frame (ORF) on genome segment 9. NS4 is a protein that is expressed as early as 4 h post-infection and localizes to both the cytoplasm and the nucleus, particularly the nucleolus. All orbiviruses sequenced to date have been shown to contain a fully functional NS4 ORF, except SCRV in which the NS4 ORF is interrupted by a stop codon (Belhouchet et al., 2011). Immunofluorescence analyses have shown the presence of NS4 in the cell membrane during late stages of infection in bluetongue virus-infected mammalian cells. NS4 associates with lipids and, like other viruses which make use of lipid pathways such as hepatitis C virus (Feld, 2012), NS4 is likely to be involved in virus exit and possibly re-infection. The defective NS4 ORF of SCRV may explain in part why this virus is unable to infect cell lines other than those derived from ticks (Belhouchet et al., 2011), though not how it can transfer from one tick cell line to another. However, four types of virus particles are known for orbiviruses. These include whole intact virus particles (having the two outer capsid proteins), infectious subviral particles (generated by the effect of proteases on whole particles), transiently-enveloped particles and core particles (which have lost the outer capsid proteins) (Mertens et al., 1987; Belhouchet et al., 2011). Core particles are known to be able to infect arthropod-derived cell lines, bypassing the requirement for the NS4 protein as part of the transiently enveloped particle (Mertens et al., 1987). Moreover, many tick cells *in vitro* have a tendency to be strongly phagocytic (authors' unpublished observations) and could actively take up virus particles.

Since tick cell lines are increasingly being used for isolating and propagating viruses and bacteria, the possible consequences of the presence of endogenous tick viruses (ETV) on co-infection with exogenous microorganisms must be considered.

## Co-infections in ticks and tick cell lines

In nature, ticks may be infected with more than one pathogen, but little is known about the interactions between different pathogens co-infecting the same tick, nor about the interactions between co-infecting pathogens and endosymbiotic bacteria (Ginsberg, 2008). If, as it seems likely, many ticks harbor one or more endogenous tick viruses (ETV), these might be expected to interact in some way with single or multiple pathogen infections within the host tick. Such interactions could be direct interactions between microorganisms, or indirect interactions at the cellular level through modulation by the ETV of the host cell response to the coinfecting pathogen.

Arboviruses are known to cause lifelong infections in ticks, and may be transmitted transovarially albeit at a low level (Labuda and Nuttall, 2004). Similarly, arbovirus infections in tick cell lines generally cause little or no cytopathic effect (CPE) and persist for extremely long periods (Leake et al., 1980; Leake, 1987; Bell-Sakyi et al., 2012). Tick-borne bacteria and protozoa may also persist within the host for extended periods—*Ehrlichia ruminantium* can survive within *A. variegatum* ticks for 15 months [Ilemobade 1976 cited by Camus et al. (1996)], and some species of *Babesia* are transovarially transmitted and can persist through multiple generations of ticks even when they are feeding on non-susceptible hosts (Chauvin et al., 2009). Such persistent infections, if heavy, may adversely affect tick longevity but generally do not prevent feeding or oviposition, whereas *in vitro* pathogenic bacteria of the genera *Anaplasma, Ehrlichia* and *Rickettsia* usually destroy the host cell. In contrast, the endosymbiont *R. peacockii* has little adverse effect on cells of its natural host *D. andersoni*, but when transferred to cells of different tick species, the bacterium can cause cell death (Kurtti et al., 2005). When the ETV SCRV was transferred from IDE8 cells to RSE8 cells via ISE6 cells, no CPE that could be ascribed to the virus was detected in either of the recipient cell lines. Similarly, the presence of SCRV in RA243 cells does not appear to have any adverse effect, as the cell line in our laboratory is in good health, can be subcultured at fortnightly intervals and is currently at passage 355 (authors' unpublished observations).

In order to begin to elucidate the possible role of ETV in modulating co-infections with arboviruses and intracellular bacteria at the cellular level, it is necessary to turn to research on EVE and endogenous viruses of other arthropods, in particular mosquitoes which share a haematophagous lifestyle with ticks, and *Drosophila*, the most-studied arthropod to date.

## Endogenous viruses may modulate arbovirus infection in insects

EVE in arthropod genomes have been identified in both insects and ticks. Those found in insects belong to or are related to the genera *Nucleopolyhedrovirus* (dsDNA), *Nudivirus* (dsDNA), *Totivirus* (dsRNA), *Partitivirus* (dsRNA), *Seadornavirus* (dsRNA), *Cripavirus* (+ssRNA), *Flavivirus* (+ssRNA), *Phlebovirus* (−ssRNA), *Nairovirus* (−ssRNA), *Lyssavirus* (−ssRNA), and *Thogotovirus* (−ssRNA) (Crochu et al., 2004; Taylor and Bruenn, 2009; Katzourakis and Gifford, 2010; Liu et al., 2010; Feschotte and Gilbert, 2012). Once integrated into the host genome, the viral genome becomes a “fossil” DNA copy that is unlikely to give rise to a replicating virus.

Endogenous replicating viruses identified in arthropod-derived cell lines belong to a number of families. Examples of these include the C6/36 densovirus (*Parvoviridae*; *Densovirus*), cell fusing agent virus (CFAV) (*Flaviviridae*; *Flavivirus*) and *Aedes pseudoscutellaris* reovirus (ApRV) (*Reoviridae*; *Dinovernavirus*) in mosquito cell lines, SCRV (*Reoviridae*; *Orbivirus*) in tick cell lines, and *Drosophila melanogaster* totivirus (*Totiviridae*; *Artivirus*) in a *Drosophila* cell line (Attoui et al., 2001, 2005; Crochu et al., 2004; Wu et al., 2010; Zhai et al., 2010).

An endogenous arthropod virus may or may not be species-specific. For example, a densovirus (ssDNA) derived from *Armigeres* mosquitoes killed *Aedes albopictus* C6/36 cells (which are persistently infected with C6/36 densovirus), while many other densoviruses persistently infect specific mosquito cell lines (Zhai et al., 2008) in the absence of CPE. Replication of endogenous viruses in specific cell lines may significantly reduce replication of other viruses. CFAV, isolated from *Aedes aegypti* cells, interferes with replication of other flaviviruses during co-infection. When mosquito cells were infected by CFAV prior to co-infection with another flavivirus such as yellow fever virus (YFV) 17D, relatively low copy numbers of YFV could be detected. Ultimately, YFV replication was “silenced” (authors' unpublished data). However, if the co-infection with both viruses was realized simultaneously, the effect of CFAV on YFV 17D was found to be minimal.

ApRV persistently infects the mosquito cell line AP61 (Attoui et al., 2005). Real-time PCR indicated that there were only ~6 ApRV particles per cell. Treatment with an innate immune modulator such as 2-amino purine (kinase inhibitor) increased by 10-fold the number of ApRV particles per cell, indicating that the arthropod innate immune system controlled levels of virus replication, probably through the RNAi pathway. In contrast, when ApRV was used to infect C6/36 cells which have a defective RNAi pathway (Morazzani et al., 2012), virus titers reached ~5 × 10^4^ viral particles/cell. Moreover, an exogenous virus may have unexpected effects on replication of an endogenous virus as seen with infection of *Aedes albopictus* cell lines. Following Liaoning virus (LNV) infection of C6/36 cells, significantly large amounts (milligram quantities) of the C6/36 densovirus were generated alongside similar quantities of LNV (Attoui et al., 2006), suggesting that dsRNA viruses in particular possess viral silencing suppressor proteins which partly counteract the host antiviral RNA interference (RNAi) mechanism.

The presence of ApRV in AP61 cells may also explain why these cells may not support replication of particular viruses. When Saboya virus (SABV, genus *Flavivirus*) was used to infect AP61, relatively low copy numbers of SABV were detected (authors' unpublished observation). Like endogenous viruses, endosymbiotic bacteria may also interfere with the replication of an exogenous virus. Dengue virus replication, dissemination and transmission were suppressed in *Aedes aegypti* mosquitoes infected with *Wolbachia* (Eleftherianos et al., 2013). Higher levels of Cecropin and Defensin were depicted in the *Wolbachia*-infected mosquitoes. There was an up-regulation of various Toll pathway genes, which was linked to a prevention of oxidative stress in the mosquitoes. Up-regulation of the Toll pathway may therefore be a mechanism by which mosquitoes control particular virus infections (Bian et al., 2010; Pan et al., 2012). These findings contrasted with the situation in *Aedes albopictus*, where naturally-occurring *Wolbachia* infection did not protect mosquitoes from being infected with dengue virus, while specific exogenous *Wolbachia* strains inhibited virus transmission (Blagrove et al., 2012; Lu et al., 2012; Eleftherianos et al., 2013).

## Do endogenous viruses play a modulating role in infection of tick cells with arboviruses and bacteria?

In order to study the possible involvement of an ETV in modulation of pathogen infection in tick cells, the nature of the ETV genome and its taxonomic status need to be known. This information is necessary to understand the replication cycle and interactions of the ETV with various signaling pathways. Initial insights may be gained from ultrastructural analyses using thin EM sections of tick cells and these may reveal morphological similarities to known groups of arboviruses. Universal molecular techniques have become an essential tool for the identification of naturally occurring arboviruses in biological samples. Of particular interest are those based on broad spectrum RT-PCR approaches. There are several conventional or real-time pan-generic PCR approaches for the identification of flaviviruses (Moureau et al., 2007), orbiviruses (Palacios et al., 2011) and bunyaviruses, particularly pan-nairovirus, pan-orthobunyavirus and pan-phlebovirus PCRs (Lambert and Lanciotti, 2009). Full-length characterizations may be realized using virus sequence-independent methodologies described for dsRNA including the single primer amplification technique (SPAT, Attoui et al., 2000) or the SPAT-derived full length amplification of complementary cDNA (FLAC, Maan et al., 2007), or for ssRNA such as that combining a FLAC/SMART (Switch Mechanism At the 5′ end of RNA Template) methodology (Attoui et al., 2007).

Ultimately, silencing replication of an endogenous virus may be a practical way to generate a virus-free cell line. The replication of ApRV in AP61 cells was silenced by continuously feeding AP61 cells with ApR specific siRNA derived, *in vitro*, from the entire virus genome by treatment with recombinant Dicer. An ApRV-free AP61 cell line was hence derived that permitted replication of higher titers of SABV (authors' unpublished results). It will be interesting to apply this methodology to tick cell lines and attempt to derive ETV-free tick cells. The well-characterized SCRV represents an ideal target given the availability of the entire genome sequence (Attoui et al., 2001); this ETV infects three tick cell lines, IDE2, IDE8 and RA243 (Attoui et al., 2001; Alberdi et al., 2012) that together support the growth of a wide range of viral and bacterial pathogens (Bell-Sakyi et al., 2007, 2012).

**Figure 4:**
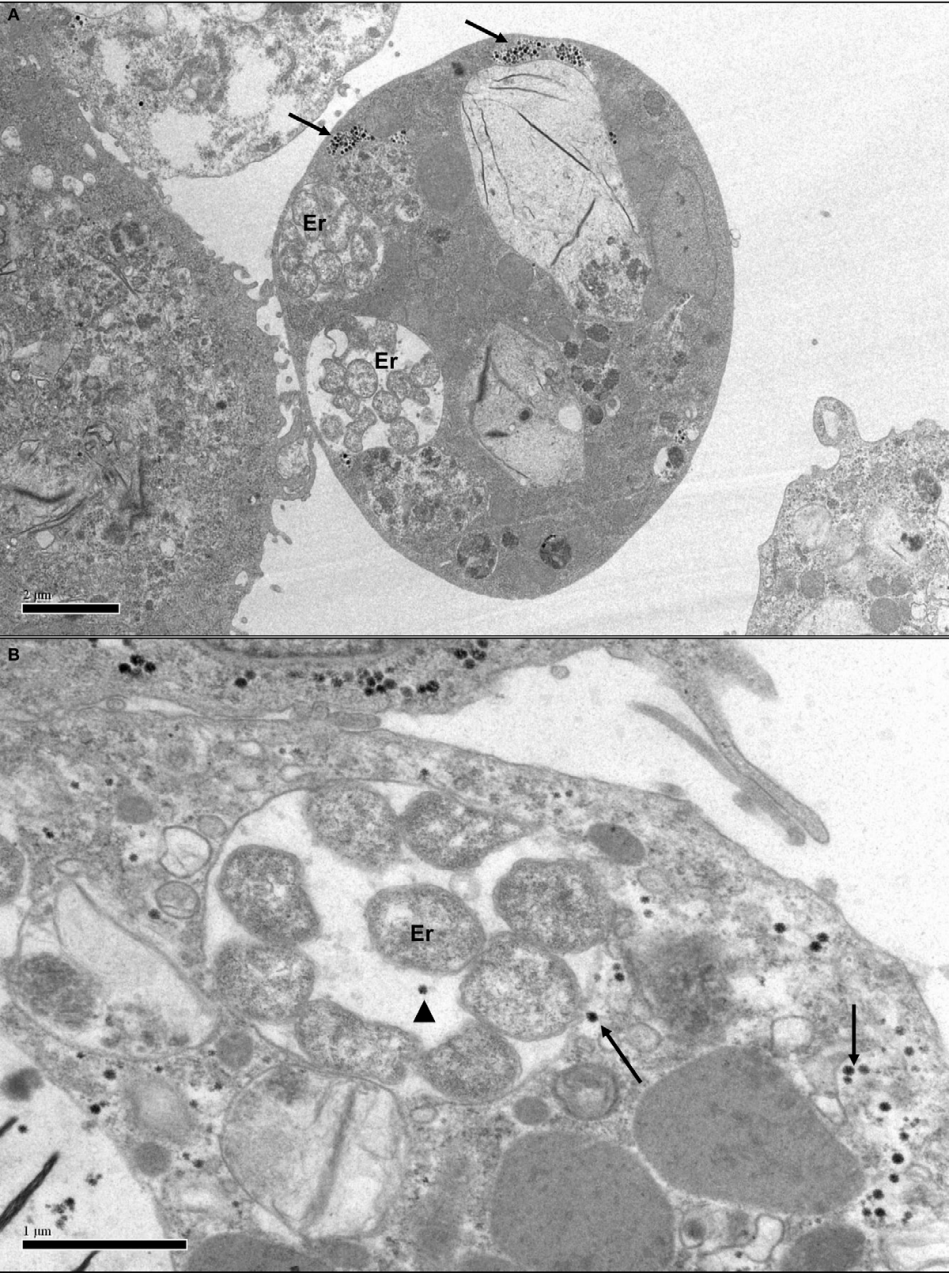
**Transmission electron micrographs of *Ixodes scapularis* cell line IDE8 infected with *Ehrlichia ruminantium*. (A)** IDE8 cell showing membrane-bound intracytoplasmic morulae containing several *E. ruminantium* bacteria (Er) co-infecting the same cell as SCRV particles in aggregates (arrows). Scale bar 2.0 μm. **(B)** IDE8 cell with aggregated and single SCRV particles, apparently even sharing the same cellular compartment as *E. ruminantium* bacteria (arrowhead). Scale bar 1.0 μm.

Comparison of ETV-infected and ETV-free tick cell lines would be an ideal model system to study the effect of the ETV on replication of various exogenous pathogens, for example SCRV and *E. ruminantium* in IDE8 cells (Figure 4), and the effect that ETV may have on up-regulating or down-regulating host cell pathways including those involved in innate immunity to microbial infection. Future work in our laboratory will focus on creation of ETV-free cell lines that will allow us to examine the role of ETV both in co-infections and potentially in cell line immortalization.

### Conflict of interest statement

The authors declare that the research was conducted in the absence of any commercial or financial relationships that could be construed as a potential conflict of interest.
